# Development of a Bioreactor-Coupled Flow-Cell Setup for 3D In Situ Nanotomography of Mg Alloy Biodegradation

**DOI:** 10.1021/acsami.3c04054

**Published:** 2023-07-17

**Authors:** Jan Reimers, Huu Chánh Trinh, Björn Wiese, Sebastian Meyer, Jens Brehling, Silja Flenner, Johannes Hagemann, Maximilian Kruth, Lidia Kibkalo, Hanna Ćwieka, Birte Hindenlang, Marta Lipinska-Chwalek, Joachim Mayer, Regine Willumeit-Römer, Imke Greving, Berit Zeller-Plumhoff

**Affiliations:** ‡ Institute of Metallic Biomaterials, Helmholtz-Zentrum Hereon, Max-Planck-Strasse 1, Geesthacht 21502, Germany; § Ernst Ruska-Centre for Microscopy and Spectroscopy with Electrons, Forschungszentrum Jülich GmbH, Jülich 52425, Germany; ⊥ Institute of Materials Physics, Helmholtz-Zentrum Hereon, Max-Planck-Strasse 1, Geesthacht 21502, Germany; || CXNS−Center for X-ray and Nano Science, Deutsches Elektronen-Synchrotron DESY, Notkestraße 85, Hamburg 22607, Germany; # Central Facility for Electron Microscopy, RWTH Aachen University, Ahornstraße 55, Aachen 52074, Germany

**Keywords:** synchrotron radiation, nano computed tomography, in situ, biodegradation, mg alloy, transmission electron microscopy

## Abstract

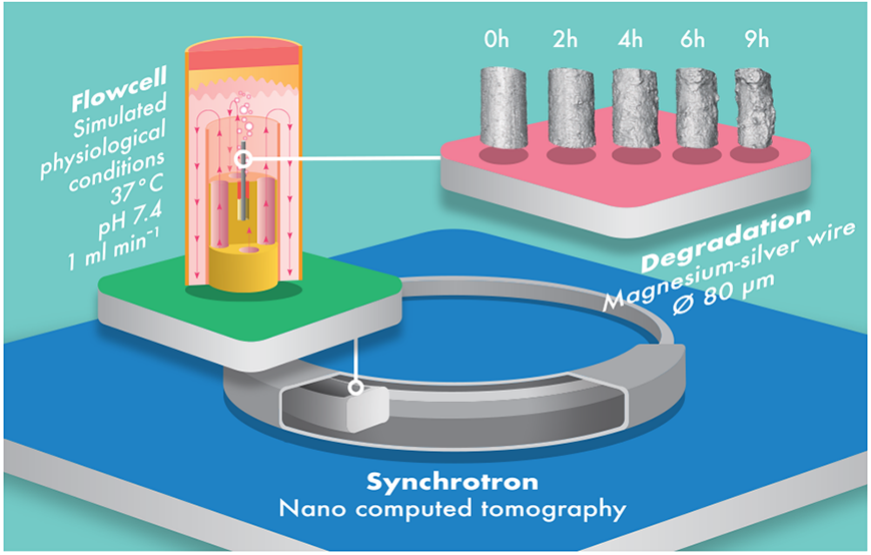

Functional materials feature hierarchical microstructures that define their unique set of properties. The prediction and tailoring of these require a multiscale knowledge of the mechanistic interaction of microstructure and property. An important material in this respect is biodegradable magnesium alloys used for implant applications. To correlate the relationship between the microstructure and the nonlinear degradation process, high-resolution *in situ* three-dimensional (3D) imaging experiments must be performed. For this purpose, a novel experimental flow cell is presented which allows for the *in situ* 3D-nano imaging of the biodegradation process of materials with nominal resolutions below 100 nm using nanofocused hard X-ray radiation from a synchrotron source. The flow cell setup can operate under adjustable physiological and hydrodynamic conditions. As a model material, the biodegradation of thin Mg-4Ag wires in simulated body fluid under physiological conditions and a flow rate of 1 mL/min is studied. The use of two full-field nanotomographic imaging techniques, namely transmission X-ray microscopy and near-field holotomography, is compared, revealing holotomography as the superior imaging technique for this purpose. Additionally, the importance of maintaining physiological conditions is highlighted by the preliminary results. Supporting measurements using electron microscopy to investigate the chemical composition of the samples after degradation are performed.

## Introduction

**Figure 1:**
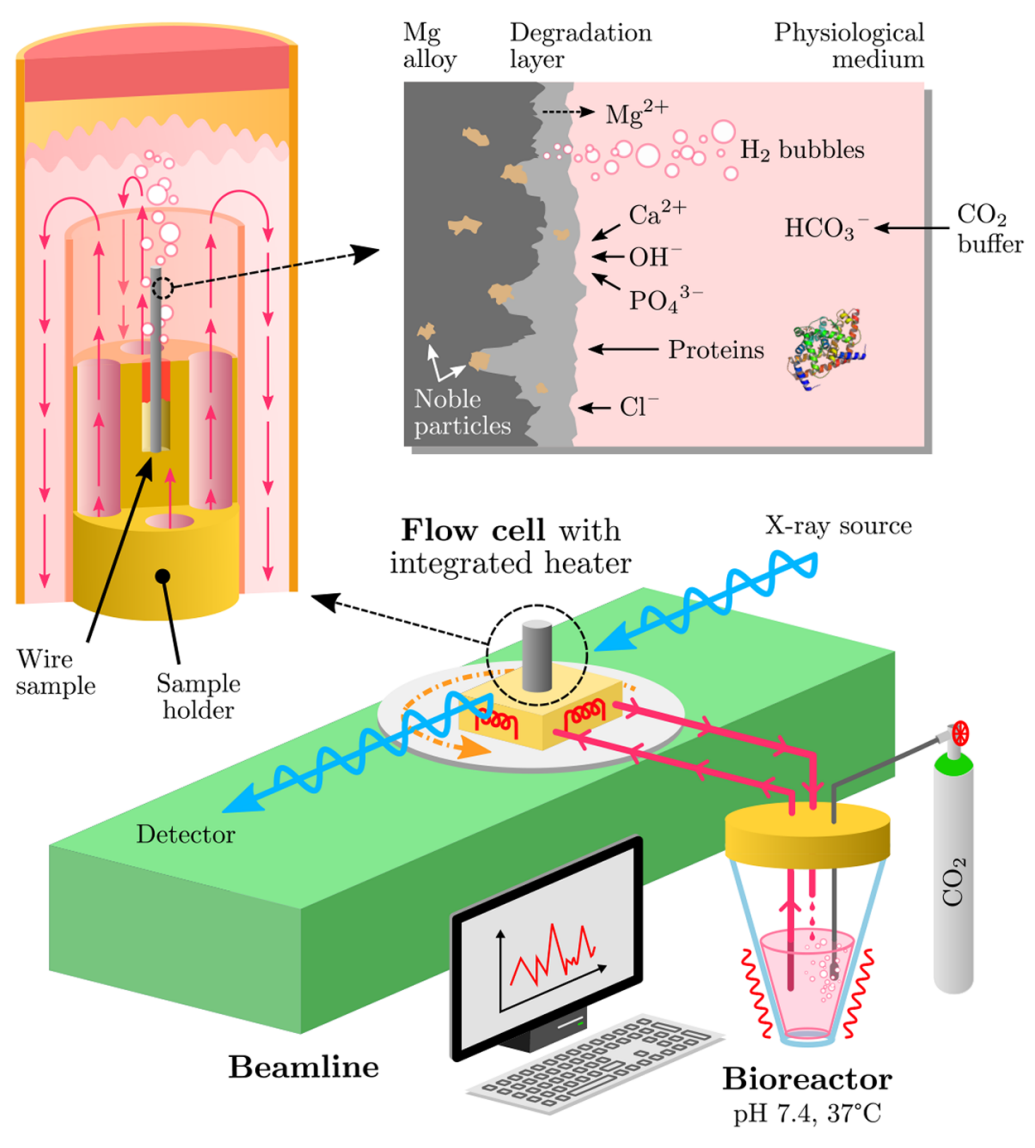
At the bottom, a schematic depiction of the flow cell setup coupled with a bioreactor integrated into an X-ray imaging beamline for *in situ* studies is shown. In the upper left corner the flow cell is enlarged showing the principal medium flow. Key interactions between the physiological medium and the Mg alloy are depicted in the upper right corner.

Multiscale *in situ* imaging of functional materials is key for understanding their dynamic properties. To this end, hard X-ray synchrotron radiation-based computed tomography (CT) can be used as an unique modality due to its nondestructive 3D nature.^1^ With the growing interest and newest developments in *in situ* measurements using CT imaging, a variety of dynamic processes in different research areas are being investigated, e.g., electrochemical processes in batteries^2^ and fuel cells,^3^ as well as material deformation mechanisms.^4−6^ It has been shown that 4D (spatial and temporal) imaging is particularly useful to understand complex nonlinear processes such as the biodegradation of novel magnesium (Mg) alloys meant for temporary medical implants inside the human body.^7^ Mg-based alloys are emerging as a compelling alternative to traditional implant materials like stainless steel and titanium alloys, which often necessitate a removal surgery as the risk of an implant rejection increases over time.^8−10^ By eliminating the need for a second surgery, biodegradable implants reduce health risks for the patient and financial costs for the healthcare system.^11−13^ However, high degradation rates (DR) of Mg and its alloys limit their practical application as implant materials. This is because the implant may degrade too quickly before the healing process of the surrounding bone or blood vessel is complete.^14^ Moreover, as the degradation of Mg-based implants changes the local chemical environment, controlling the degradation rate is pivotal to ensure a favorable tissue response. Figure 1 shows some of the key interactions considered for the biodegradation of Mg alloys exposed to physiological conditions. To be able to predict and tailor the material behavior in the future, it is necessary to elucidate the correlation between the microstructural features of the alloy, such as precipitates, the involved transport processes guided by the porosity of the forming degradation layer on the surface, and the dynamic process of the implant degradation. To this end, hard X-ray synchrotron radiation-based nanocomputed tomography (SRnanoCT) is a uniquely suitable technique, as it enables the visualization of precipitates^15,16^ and pores,^17^ as well as the overall implant morphology.^18^

In this work, a novel flow cell setup is presented, dedicated to perform, for the first time, *in situ* SRnanoCT imaging of the *in vitro* degradation of Mg-4wt%Ag (silver) wires under physiological conditions. The flow cell is integrated into the setup of the nanotomography endstation at the Imaging Beamline (IBL) P05 operated by Hereon at the storage ring PETRA III at the Deutsches Elektronen-Synchrotron (DESY) in Hamburg, Germany, where SRnanoCT experiments are conducted.^19^ A schematic depiction of the flow cell integrated into the IBL is shown in Figure 1. The flow cell design and imaging setup is based on several requirements:

1. The flow cell must be able to be integrated into the IBL, which includes the ability for rotation by 180° with high stability, low absorption of hard X-ray synchrotron radiation, and sterilizability.
2. The *in vitro* degradation conditions have to mimic the physiological conditions as close as possible. This includes the use of complex cell culture media, and temperature (37 °C) and pH control (7.4).^20^
3. The flow cell must enable setting specific hydrodynamic conditions around the sample.
4. The imaging setup must provide a sufficiently high resolution and contrast in order to resolve all features of interest in the sample.

As a model material system, Mg alloyed with 4 wt % silver (Mg-4Ag) is investigated in the shape of wire pieces with 80 μm diameter and 2 mm length. Due to its excellent balance of relatively low DR and good mechanical properties, homogenized Mg-4Ag is considered to show a high potential as antibacterial^21^ and biodegradable implant material.^22^ The features of interest, which need to be resolved for the understanding of Mg alloy biodegradation, are precipitates, pores, and the early stage formation of a degradation layer (DL).

Full-field SRnanoCT techniques available at IBL include transmission X-ray microscopy (TXM) with Zernike phase contrast^19^ and near-field holotomography (NFHT),^23^ both of which can be used to study Mg alloys.^1^ These techniques differ in several aspects, such as contrast mechanism and resolution. Therefore this study compares both TXM and NFHT with respect to their imaging and segmentation quality, the two major factors most critical for the qualitative and quantitative evaluation of the biodegradation process.

As *in situ* SRnanoCT does not provide information on the chemical composition about the DL and precipitates, supportive measurements with the aid of transmission electron microscopy (TEM) and energy-dispersive X-ray spectroscopy (EDX) complete our study.

In the following, the design of the flow cell experimental setup is described, which fulfills all the aforementioned functional requirements. The functionality of this novel flow cell setup is subsequently evaluated and verified by experimental findings and simulations. The qualitative and quantitative SRnanoCT data regarding the biodegradation of Mg-4Ag are shown subsequently.

## Experimental Section

### Materials

Mg-4Ag wires with a diameter of 80 μm are manufactured at the Helmholtz-Zentrum Hereon in Geesthacht, Germany. Pure Mg (purity: 99.98%, MAGONTEC, Sydney, Australia) is melted and alloyed with 4 wt% Ag (purity: 99.99%, ESG Edelmetall-Service GmbH & Co. KG, Rheinstetten, Germany) under a protective argon + 3 vol% SF_6_ atmosphere. The melt is cast into a billet by a modified permanent direct chill casting technique and homogenized for 16 h at 450 °C.^24^ The homogenized billet is hot extruded at 425 °C with a mean exit speed of 3.1 m/min and an extrusion ratio of 1:625 into four Mg-4Ag wires with 1 mm diameter. Afterward, the wires are drawn into 80 μm thin wires using the drawing machine “EZ2.01” (Müller Engineering GmbH Co. KG, Todtenweis, Germany). In order to ease the drawing process, drawing wax is employed until the wire reached 320 μm in thickness. Each drawing pass reduces the wire area by about 20% with a drawing speed of 0.25 m/s. After every second pass, the wires are recrystallized at 425 °C for 5 min to avoid embrittlement and breakage by reducing work hardening while retaining solid-solution.^18,25^ Although microstructural analysis was not conducted in our study, we anticipate that the microstructure of our samples is comparable to that described by Meyer et al.^18^ for Mg-2Ag and Mg-6Ag wires with 250 μm diameter. The material used in this study was identical to that used in a further study by Meyer et al.^26^ where no precipitates were present after heat treatment. Despite the difference in diameter, no significant difference in grain size is expected for wire diameters below 500 μm.^27^ The grain sizes in our study are expected to fall within the approximate range of 35 to 45 μm.^26^

### Bioreactor-Coupled Flow Cell

The flow cell shown in Figure 1 is coupled with a “Vario 500 Mini” bioreactor and a “TL/ 15EAD” peristaltic pump (MDX Biotech GmbH, Nörten-Hardenberg, Germany). The pH level of the degradation medium is adjusted by injection of CO_2_ gas (CANgas, purity: 99.995%, Messer Schweiz AG, Lenzburg, Switzerland). The self-built flow cell made from polyetheretherketone is located 2 m away from the bioreactor; hence, a 35 W TO220-type resistor (Bourns Inc., Riverside, USA) attached to a copper plate serves as integrated heating solution. The temperature controller “TC0806-RS232” and the proprietary software “TCCOM” (CoolTronic GmbH, Beinwil am See, Switzerland) are used to control the heater.

### Simulation of Fluid Flow in Setup

The fluid flow in the flow cell is simulated using COMSOL Multiphysics 6.0 (COMSOL AB, Stockholm, Sweden). The computer aided design of the fluid space within the flow cell is imported in Comsol and the stationary solution for laminar flow with different inflow rates is determined. To this end, the inflow rates at the inlet and outlet are set with a pressure condition of *p* = 0 Pa. The fluid properties are set to those of water (built-in in Comsol). The maximum flow velocity is determined using a cut plane positioned at the approximate height of measurement 0.6–0.8 mm above the glue position of the wire.

### *In Situ* Full-Field Nanoimaging

At the nanotomography endstation P05 operated by the Helmholtz-Zentrum Hereon at the storage ring PETRA III (DESY, Hamburg, Germany),^28^ Mg-4Ag wires are imaged *in situ* using the full-field SRnanoCT techniques TXM and NFHT. By a Si{1 1 1} double crystal monochromator the beam energy is monochromized to 11 keV. The acquisition of one full set of projections takes approximately fifteen to thirty minutes. A Hamamatsu Photonics C12849–101U camera (Hamamatsu, Japan) with a sCMOS chip with 6.5 μm physical pixel size and a 10 μm Gadox scintillation layer is used as the detector system.

In case of TXM, a similar setup as described in^17^ is used. A Fresnel zone plate (FZP) with an outermost zone width of 60 nm and a diameter of 250 μm served as the objective lens. Zernike phase contrast imaging is realized by placing phase rings in the back-focal plane of the FZP.^29^ The FOV of the setup is 100 μm and 2× 180° scans are stitched together to achieve 360° scans. *In situ* NFHT is implemented according to^23^ with a FZP having an outermost zone width of 50 nm and a diameter of 300 μm at a sample-to-FZP distance of 23.1 mm. Both setups have a sample-to-detector distance of approximately 20 m. All optics are designed and fabricated in the X-ray Optics and Applications group of the Paul Scherrer Institut (Villigen, Switzerland).

### *In Vitro* Mg Wire Biodegradation during SRnanoCT

For imaging, a 1 mm long piece is cut from a Mg-4Ag coil (80 μm diameter) using a ceramic knife. The wire sample is glued into the sample holder using the UV active glue, easyform LC gel (DETAX GmbH, Ettlingen, Germany). After curing of the glue and mounting the inner polyimide tube over the sample, the assembly is cleaned in an ultrasonic bath using a series of n-hexane (15 min), acetone (15 min), and 100 vol % ethanol (3 min).

As physiological degradation medium, SBF-JL2, in the form of a dual solution, is prepared under sterile conditions according to the literature.^30^ Approximately 200 mL of degradation medium is filled into the sterile bioreactor under the clean bench. While being stirred, the medium is heated to 37 °C and then the pH level is autoadjusted to 7.4. An initial reference scan is taken when the wire is sterilized using vol % ethanol at a flow rate of 2 mL/min for 15 min. Afterward, scans are taken in periodic intervals, while the Mg-4Ag wire degrades under flow at a rate of 1 mL/min. The solution volume to sample surface ratio (SV/SA) is 0.025 mL/cm^2^. The integrated heater provides a medium temperature of 37 °C. The degradation experiments used for the comparison of NFHT and TXM are carried out at room temperature. When a biodegradation experiment is finished, the sample holder with the degraded wire sample is cleaned with ethanol, exchanged, and stored inside a desiccator to avoid further degradation.

### Experimental Parameters

Mg-4Ag wire samples from the same coil, which were degraded in SBF, were imaged *in situ* with NFHT and TXM, respectively. This comparison was performed at RT with a first generation flow cell. Once NFHT was confirmed as the more suitable technique, it was tested again at 37 °C by using the second generation flow cell. An overview of the experiments is given in Table 1.

**Table 1:** Overview of Important Experimental Parameters for the Beamtime Experiments Using the Flow Cell Setup

	Experiment run		
Parameter	1	1	2
Flow cell generation	1	1	2
Degradation medium	SBF	SBF	SBF
Flow rate *Q* (mL min^–1^)	1	1	1
pH	7.3–7.6	7.3–7.6	7.4
Temperature *T* (°C)	RT	RT	37
SRnanoCT technique	TXM	NFHT	NFHT
Phase contrast	Zernike	quantitative	quantitative
Effective voxel size *s*_*v*_ (nm)	43	84	92
Time between scans (min)	120	120	35

TXM and NFHT differ most notably in their respective phase contrast mode: TXM relies on Zernike phase contrast, which is purely qualitative, while NFHT is able to provide quantitative phase contrast through an additional phase retrieval step. From phase retrieval to segmentation, each NFHT scan took at least 3–4 h longer to process than a TXM scan.

### Tomographic Image Processing and Analysis

Tomographic reconstruction is performed using a gridrec algorithm in TomoPy pipelines^31^ with a binning factor of 2 in all directions. Similar effective voxel sizes of the reconstructed tomograms are achieved for TXM and NFHT: 43 nm and 84–92 nm, respectively.^19,23,29^ In the case of NFHT, a phase retrieval step is required prior to reconstruction. Phase retrieval is performed from a single distance measurement using an iterative projection algorithm described in the literature.^32^ All image processing is performed on the Maxwell computing cluster at DESY.

After reconstruction, the TXM and NFHT tomograms are filtered using an iterative nonlocal means filter (control parameter α = 0.75) according to the literature^33^ or a 2D anisotropic diffusion filter (50 iterations, smoothing level 2) implemented in Avizo 2021.1 (FEI SAS, Thermo Fisher Scientific Inc., Massachusetts, USA), respectively. The filtered tomograms are segmented using Avizo 2021.1. The NFHT and, where applicable, the TXM tomograms are segmented *via* thresholding and region-based watershedding. The majority of TXM tomograms are segmented semimanually by segmenting every 10th tomographic slice by hand filling in the gaps between slices by label interpolation. Following segmentation, the tomograms are registered on another with the initial tomogram of the nondegraded sample as a reference. Then, the registered tomograms are resampled and cropped.

Image analysis and the calculation of the contrast-to-noise ratio (eq 1) and DR are performed using Fiji/ ImageJ.^34^
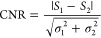
1where *S*_1/2_ is the signal and σ_1/2_ is the respective noise. The surface area *A*_0_ of the nondegraded sample is determined using a Matlab script.^35^

### Electron Microscopy and Sample Preparation

Electron microscopy experiments of a Mg-4Ag wire sample degraded in SBF are performed at the Ernst-Ruska center in Jülich. An approximately 100 nm thin TEM lamella is prepared using the Dual-FIB workstation FEI Helios NanoLab 400S (Thermo Fisher Scientific Inc., Waltham, Massachusetts). Carbon is used as a protective layer for the region of interest by both electron and ion beam deposition. At 30 kV and 5.5 nA cross-sectional trenches are milled above and below the ROI by an Ion beam (Gallium). The TEM lamella is further prepared by edge cleaning and undercutting and transferred to a copper grid by the use of a micromanipulator needle. Step wise thinning of the TEM lamella is performed until a thickness of under 100 nm is reached. A final polishing at 2 kV and 47 pA is done. HAADF STEM imaging and EDX analyses shown in Figure 7 are conducted with the aid of probe-corrected FEI Titan G2 80–200 operated at 200 kV^36^ and Velox Software (Thermo Fisher Scientific Inc., Waltham, Massachusetts). Quantification of EDX data in Table 2 is corrected for absorption, using a sample thickness of 100 nm and applying the Brown-Powell model for the ionization cross section.

**Table 2:** Deconvoluted Values of Elemental Content Given in at.% Taken from Corresponding ROIs Marked in Figure 7a–d.

	Region			
Element	a	b	c	d
C	7.49 ± 2.30	20.49 ± 4.04	40.77 ± 5.80	64.88 ± 12.04
O	5.58 ± 2.03	57.16 ± 16.00	39.67 ± 9.66	13.26 ± 4.57
Na	0.00 ± 0.05	3.53 ± 0.98	3.27 ± 0.79	0.00 ± 1.22
Mg	86.09 ± 31.13	14.01 ± 3.90	4.15 ± 1.01	1.24 ± 0.96
P	0.08 ± 0.04	2.94 ± 0.80	5.38 ± 1.26	1.43 ± 0.76
Cl	0.06 ± 0.03	0.62 ± 0.17	0.20 ± 0.05	0.00 ± 0.60
Ca	0.03 ± 0.02	0.70 ± 0.17	6.29 ± 1.21	1.35 ± 0.51
Ag	0.68 ± 0.22	0.55 ± 0.13	0.27 ± 0.05	17.84 ± 3.35

## Results and Discussion

### Flow Cell Design

**Figure 2:**
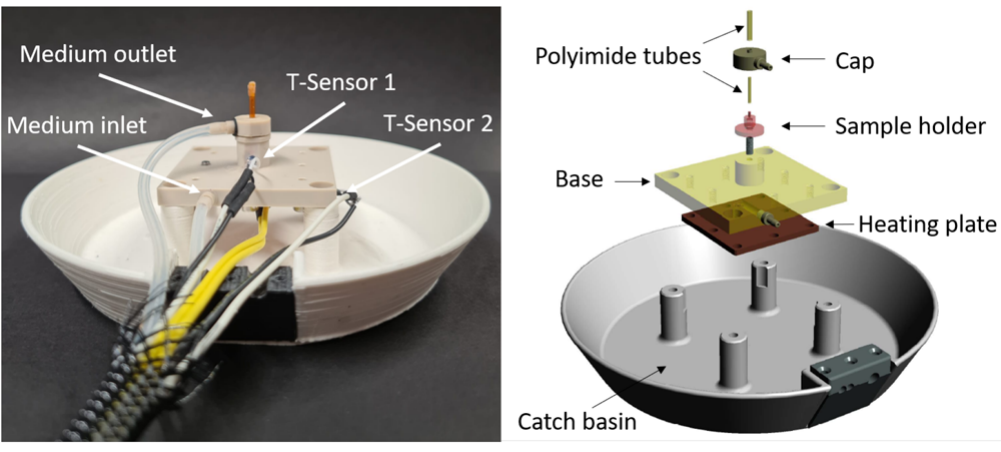
Second generation of the flow cell. A wire specimen was mounted inside the polyimide tubes on the sample holder. The sample holder has a height of 7.2 mm and a diameter at the base of 10 mm. The two hoses are the inlet and outlet for the medium. Yellow wires are connected to the copper heating plate underneath the sample stage. The pairs of black and white wires are connected to temperature sensors, one at the copper plate and one directly in front of the sample location.

The central element of the flow cell setup is a specialized sample environment that permits the *in situ* imaging of the biodegradation processes of the Mg alloy wire. There are two generations of the flow cell, with the second generation including the integration of a heating unit. In the following, the second generation flow cell shown in Figure 2 is described in detail. The flow cell consists of four parts: a catch basin, the base unit with an integrated heater, the sample holder, and a cap. The catch basin has four bores which are used to bolt the flow cell onto the rotation axis of the nanotomography setup. The black part on the rim of the catch basin is the cable relief. At the center of the catch basin is the base unit an inlet for media. Not shown is the integrated heating plate below the base unit, which is connected to a power source *via* electrical cables. The heating plate is made from copper and is separated from the degradation medium by a thin polyimide sheet to prevent any contamination of the medium. The base unit houses two temperature sensors, one monitors the temperature of the heater (T-sensor 1) and the other of the medium (T-sensor 2). The sample holder is screwed tightly onto the base unit in order to avoid leakage. The same holds for the cap, which is screwed onto the sample holder and has an outflow grommet for the cell culture medium harvest. The flow cell inlet and outlet are connected to a bioreactor to maintain physiological conditions during testing.

Except for the catch basin, all major parts are machined from polyetheretherketon (PEEK) because of its good mechanical and thermal properties and chemical stability permitting the sterilization of the flow cell using 70% ethanol solution.^7^ The parts inside the beam path are made of polyimide which is low-absorbant toward synchrotron radiation.^37^ The flow cell is designed such that during the 180° rotation, none of the cables and silicone tubes can obstruct the beam and have as little influence on the sample stage as possible (see Video S1).

### Simulating Physiological and Hydrodynamic Conditions

An integral function of the flow cell setup is the simulation of physiological conditions. By coupling the flow cell with a bioreactor, physiological media can be stored and the pH controlled. In the current work, simulated body fluid (SBF) prepared according to^30^ (SBF-JL2) is used, but using other immersion media, such as cell culture media supplemented with proteins would be possible too. The pH of the medium is adjusted in the bioreactor using CO_2_ gas, which is injected directly into the media at bodylike temperatures (37 °C) until a pH of 7.4 is reached.

**Figure 3:**
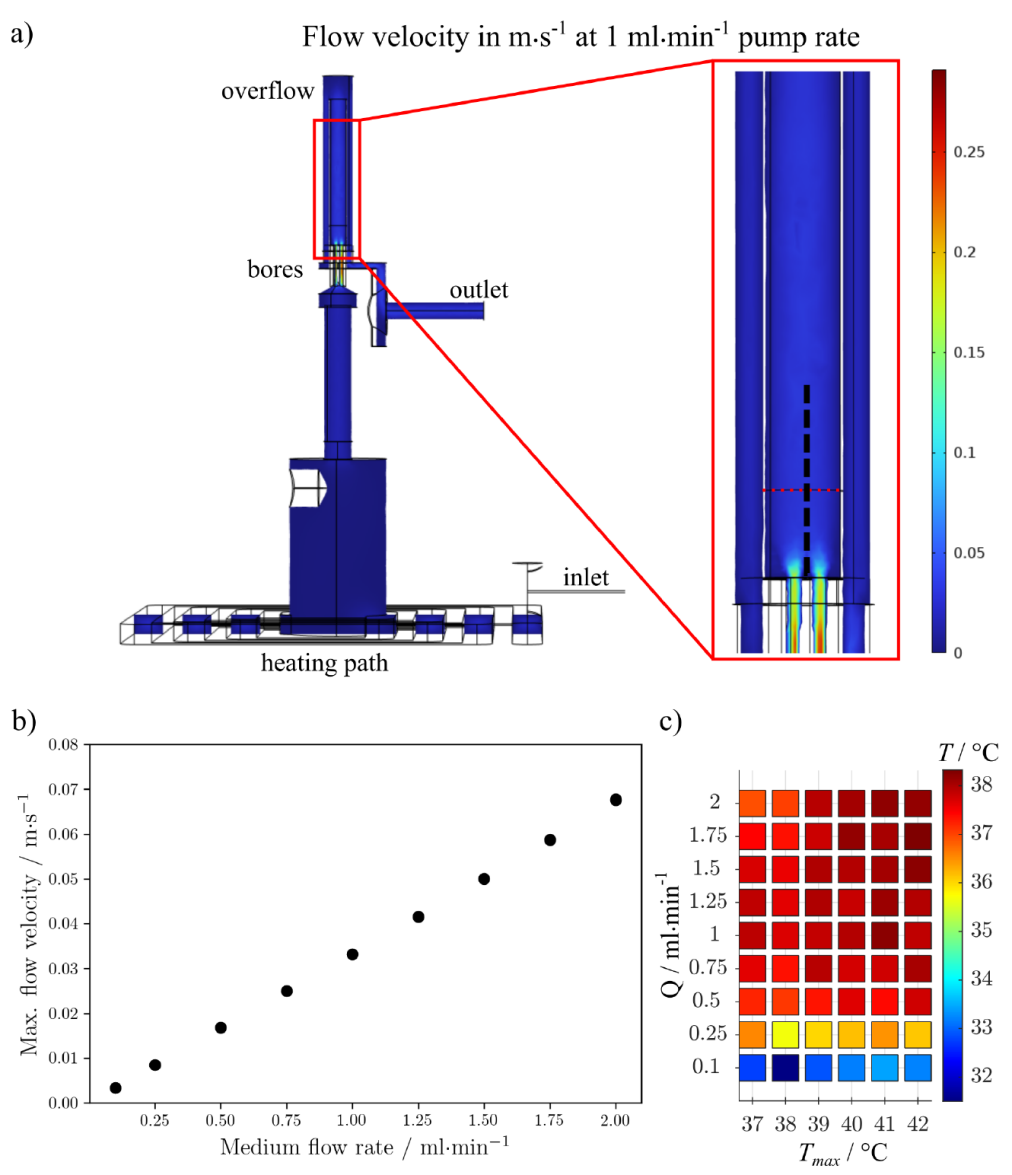
(a) Simulated flow velocity at a flow rate of 1 mL·min^–1^ in the flow cell. The dotted red line in the inset highlights the appoximate height at which the sample was measured. The sample itself is indicated by a dashed black line. (b) Maximum flow velocity in the inner polyimide tube evaluated at the measured sample height for different pump flow rates. (c) Experimental data of the integrated heater of the flow cell. Measured temperatures are *T* inside the flow cell for different flow rates *Q* and maximum temperatures *T*_max_.

Many *in vitro* Mg alloy degradation studies^38,39^ are performed under physiological conditions, however, the medium remains quasi-static. This is considered one of the reasons for the discrepancy between *in vivo* and *in vitro* degradation.^40,41^ Hence, the establishment of hydrodynamic conditions is another key consideration for the flow cell setup. Using a peristaltic pump, the medium is fed to the flow cell at different flow rates. The medium enters the sample chamber as shown in Figure 1 and a flow surrounding the Mg alloy wire is established. The presence of a reservoir (200 mL) within the bioreactor ensures a high buffer capacity and minimizes the likelihood of reaching saturation or depletion of essential components.^20^ As a result, the influence of the electrolyte volume is considered negligible. In order to characterize the hydrodynamic conditions, a simulation of the flow is shown in Figure 3a where the flow velocity within the flow cell is shown for a flow rate of 1 mL/min. The maximum flow rate in the inner polyimide tube at the measured sample height is evaluated in Figure 3b. Overall, the flow is laminar, with a strong increase in flow velocity only within the fine bores. As the simulation shows, the flow velocity surrounding the sample can be controlled linearly by adjusting the flow rate. Due to the distance between bioreactor and sample (approximately 2 m), the medium temperature drops down to room temperature (RT) when being pumped to the sample stage. To resolve this and preserve a physiological temperature of 37 °C, the second generation of the bioreactor-coupled flow cell setup contained an integrated heater. The heating characteristics for different medium flow rates is shown in Figure 3c. The maximum temperature *T*_max_ is measured by T-sensor 1 which is directly attached to the integrated heater. After thermal equilibration, the medium temperature *T* is measured by using a standalone temperature sensor placed inside the inner polyimide tube. The *T* values at the sample position are lower than *T*_max_ since the medium is heated only while flowing across the base of the flow cell, where the heating unit is connected. Consequently, the temperature increases with flow rate *Q* as the medium flows more quickly to the sample and sensor. Therefore, it is not possible to achieve physiological temperatures at flow rates below 0.25 mL/min. Nevertheless, for a wide range of flow rates *Q* physiological temperatures are reached while *T*_max_ is in a safe range for protein-containing media.^42^

### Tomographic Imaging at the Nanoscale

Once the flow cell is established, the most suitable SRnanoCT imaging method needs to be identified in terms of its ability to generate high-resolution tomographic images of the degrading Mg-4Ag wires under *in situ* conditions with sufficient image contrast to identify key features in the samples. In the following, both techniques are compared with regard to their image contrast, resolution of microstructural feature resolution, and segmentability.

#### Image Contrast

**Figure 4:**
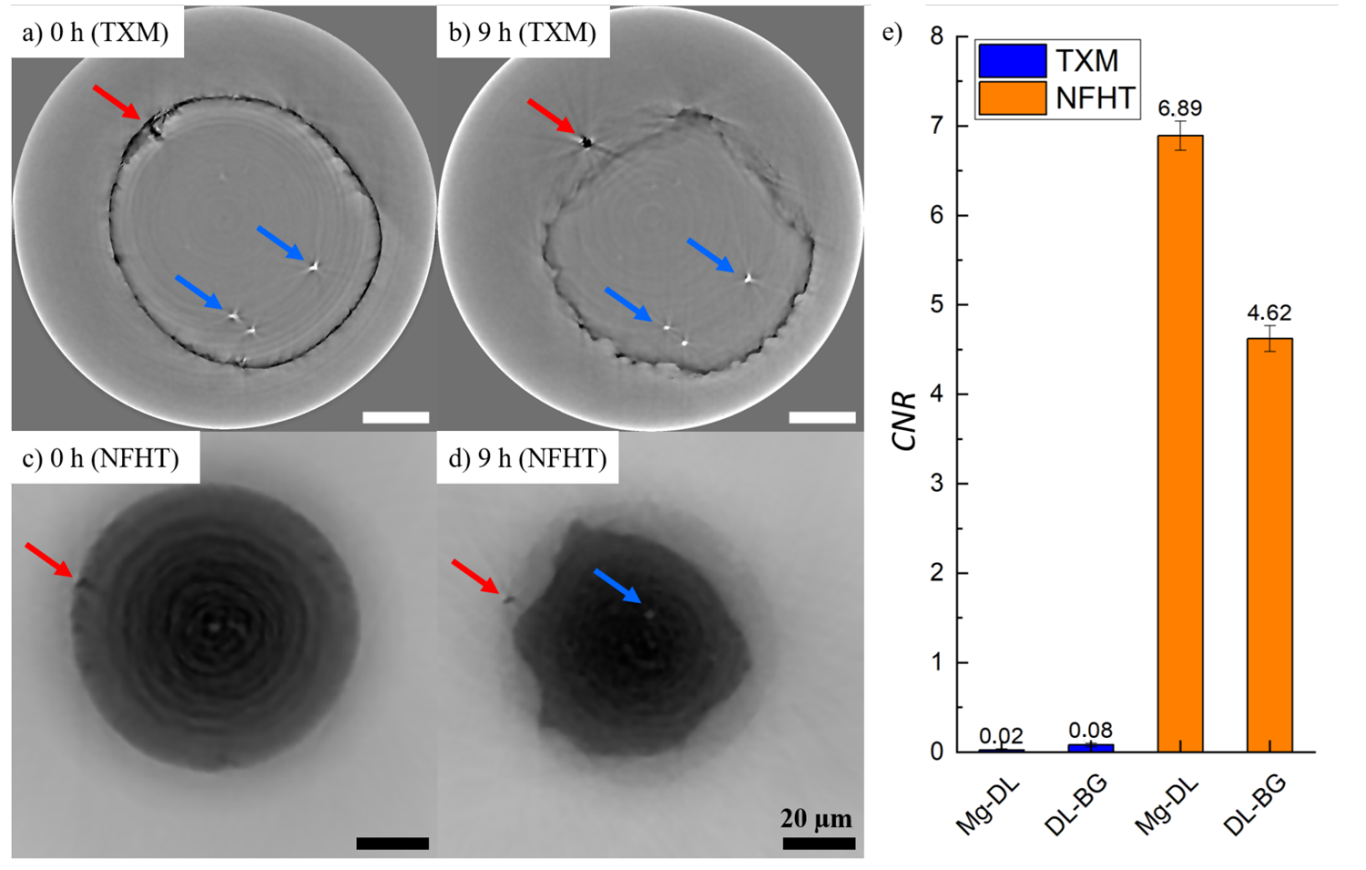
(a, b) TXM and (c, d) NFHT slices of 80 μm diameter Mg-4Ag wires degraded in SBF at RT for 9 h. Microstructural features are indicated by red arrows (possibly precipitates or impurities) and blue arrows (pores). The tomograms are filtered using an iterative non local means filter. (e) Plots of the CNR for unfiltered TXM and NFHT tomograms between bulk magnesium alloy (Mg) and degradation layer (DL) as well as DL and background (BG). The values above each bar represent the absolute CNR value, and the error bars represent the standard deviation.

The filtered tomographic slices of both TXM and NFHT measurements are shown in Figure 4a–d. Both techniques produced a strongly differing image contrast. The background (BG) in TXM has a similar grayscale value like the residual material which appears to be delineated from the background by a ring of dark greyscale values. In NFHT images, however, the dark-appearing residual material is clearly distinguishable from the light background. Additionally, a degradation layer is discernible in NFHT images, while it is hardly identifiable in the TXM images. In general, the NFHT tomographies provide superior contrast between residual material and background than TXM which is reflected by the CNR.^43^ As shown in Figure 4, the CNR values of the NFHT images surpass the CNR for TXM for both the contrast between residual Mg-alloy and DL as well as DL and BG.

Within all images phase and 3D reconstruction artifacts occur. Notable image artifacts are ring artifacts found in the TXM images. NFHT images contain a grayscale gradient and fringe-like artifacts which appear within the residual Mg part of the tomography. In addition, streak artifacts around precipitates and pores are seen in both imaging techniques, but predominantly in TXM tomograms. These result from the sample movement due to the high photon flux on the sample in the medium. Fringes appear due to strong phase gradients not being compatible with assumptions made for linearized phase retrieval.^16^

#### Feature Resolution

The resolution is important for evaluating the effect of the microstructure on the degradation behavior. Both imaging techniques enable high spatial resolutions below 100 nm. Due to the point spread function of the detector, resolutions in the range of 2.5 times the effective voxel size can be achieved (see Table 1).^23,29^ It is assumed that this resolution cannot be achieved here due to image artifacts. The arrows in Figure 4 indicate the supposed microstructural features: Pores that appear bright and have a size of 3 ± 1 μm are marked by blue arrows. The red arrows indicate Ag-containing precipitates, presumably Mg_54_Ag_17_,^44^ which appear dark and have an irregular shape. It appears visually that the microstructural features inside the wire sample are slightly better resolved by TXM than by NFHT. The chemical analysis of the postdegradation Mg-4Ag wires using EDX is presented in Figure 7 and Table 2 and the corresponding paragraph.

#### Segmentation

Segmentation is a necessary image-postprocessing step for the quantitative analysis of the degradation behavior. Segmenting the residual nondegraded Mg alloy and thus determining the number of voxels *N* belonging to it is of particular interest. Together with the voxel size *s*_*v*_, the volume *V* of the residual material is determined:
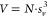
2

**Figure 5:**
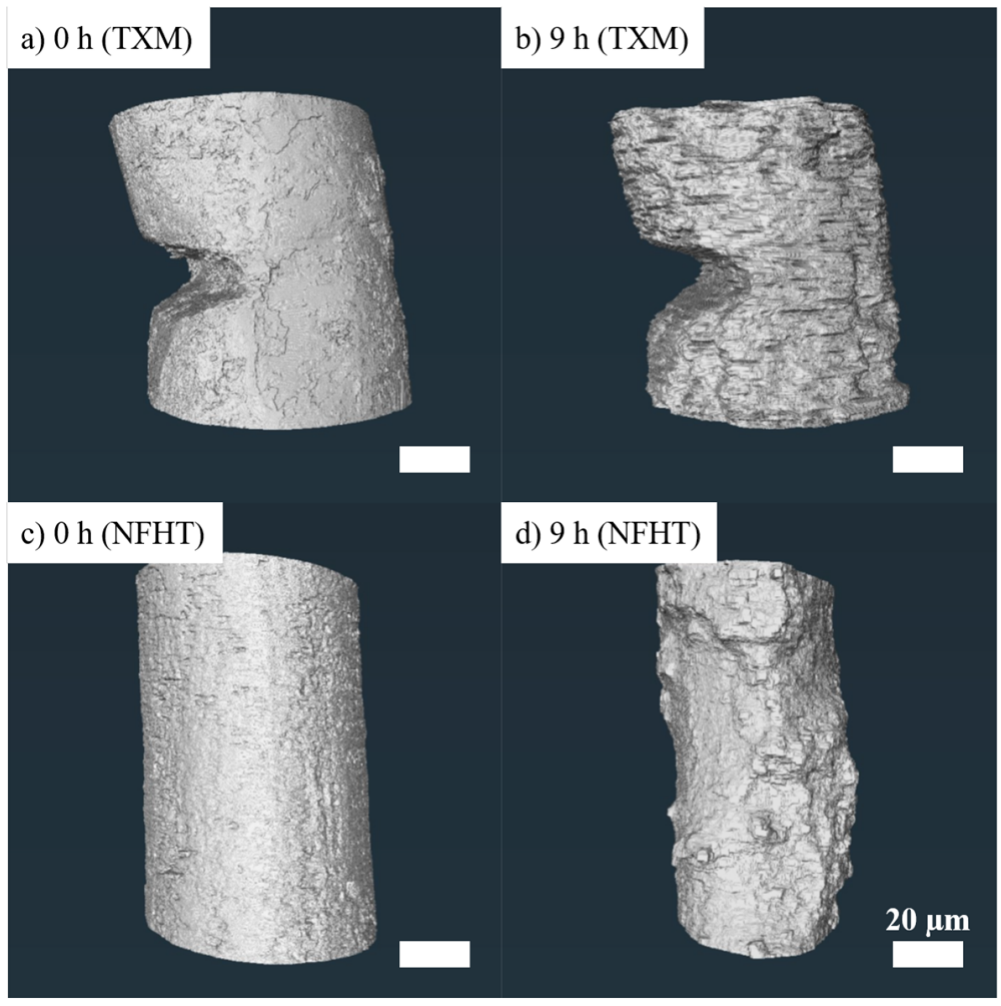
Three-dimensional isosurfaces depicting the segmentation of the remaining bulk material of Mg-4Ag wires during degradation at 0 and 9 h, utilizing two distinct imaging techniques. (a) 0 h with TXM, (b) 9 h with TXM, (c) 0 h with NFHT, and (d) 9 h with NFHT, all acquired at RT.

However, the difference in the image contrast affected the mode of segmentation strongly. Since the contrast in the TXM images is comparatively low, a time-consuming semimanual segmentation was performed (4 h). Due to the enhanced contrast, the NFHT tomographies were segmented using a combination of thresholding of grayscale values and region-based watershedding (0.5 h). The resulting segmentations of the nondegraded and degraded Mg-4Ag wires are shown in Figure 5 as three-dimensional isosurfaces. Step-like structures can be observed in the TXM isosurfaces, which are, however, not caused by the degradation of the sample: Due to inconsistencies in the manual segmentation, the subsequent interpolation created these step-like segmentation artifacts. Additionally, the large notch that can be seen in both TXM isosurfaces originates from the sample preparation and is not caused by degradation but by the imprint of a ceramic knife.

In summary, the direct comparison of TXM and NFHT images revealed that the latter is the more suitable SRnanoCT technique for imaging of the biodegradation of Mg. This finding is based on the superior image quality and segmentability. Hence, to test the second generation of the flow cell, NFHT was employed with an improved procedure. The temporal resolution was significantly increased by reducing the time between scans (see Table 1).

### Quantitative and Qualitative Analysis of Mg-4Ag Biodegradation

In the following, the tomographic images and segmentations are used to provide a quantitative and qualitative time-resolved description of the degradation behavior of Mg-4Ag, thereby demonstrating the unique data obtainable from *in situ* SRnanoCT.

Three Mg-4Ag wire samples are analyzed and compared, see Table 1. To assess the degradation behavior quantitatively, the volume losses VL and the degradation rates DR are calculated based on the segmented image data. The VL at a specific degradation duration *t* is calculated according to:
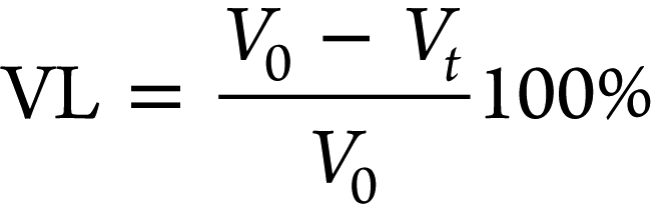
3with *V*_0_ the volume of the nondegraded sample and *V*_*t*_ the volume of the residual material, both determined using eq 2. The DR in mm a^–1^ at a specific degradation duration *t* is determined using
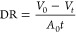
4where *A*_0_ is the surface area of the nondegraded sample.

**Figure 6:**
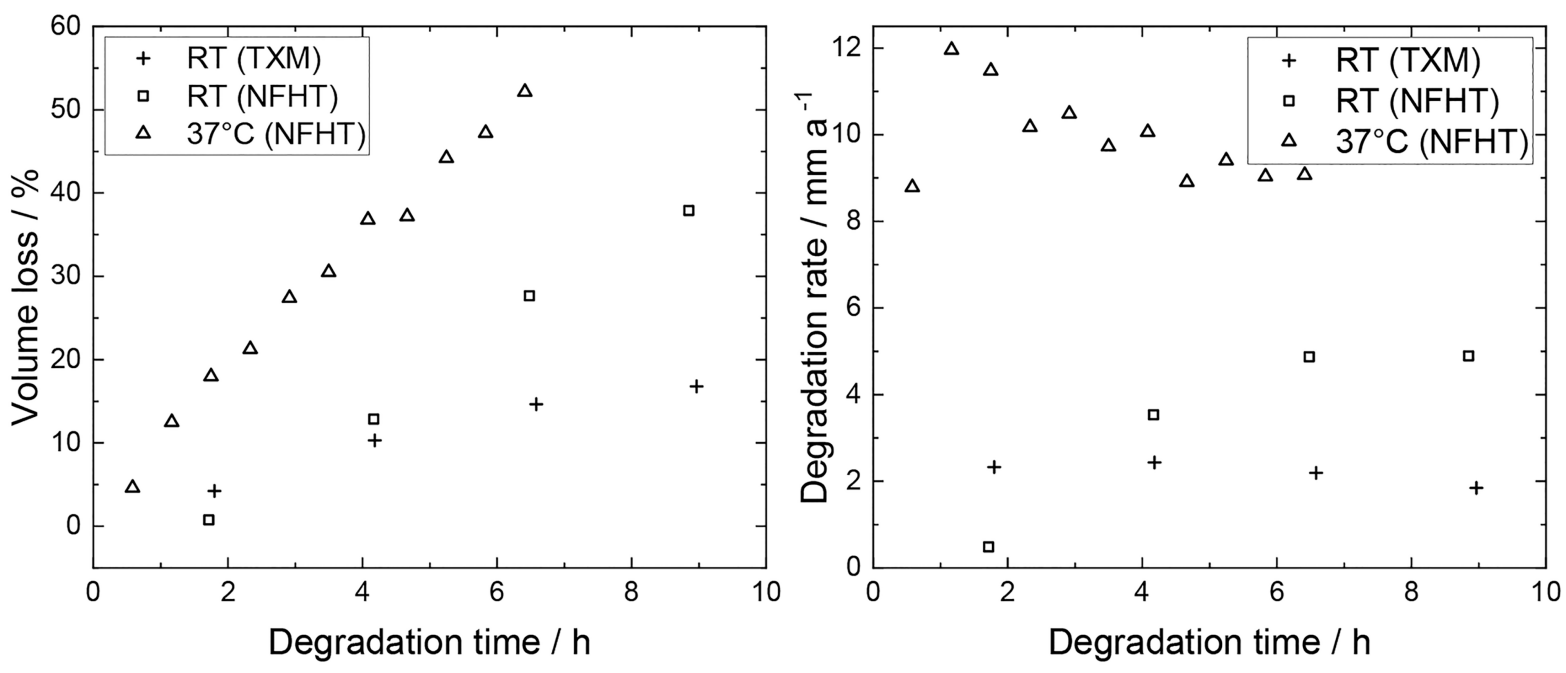
Volume losses and degradation rates of Mg-4Ag wires degraded in SBF for several hours. Results of the first generation setup at room temperature using TXM (plus) and NFHT (square) and the second generation at 37 °C using NFHT (triangle) are shown.

Figure 6 shows the degradation behavior of Mg-4Ag wires. Although the wire samples originate from the same spool and share the same processing history, their degradation behavior appears to vary strongly. The VL determined at RT differs in slope, i.e. while the VL obtained from NFHT segmentations increases linearly, the VL determined from TXM increases with a seemingly decreasing slope. These apparent differences in wire samples are in agreement with the literature, where the standard deviation of the DR of Mg-*x*Ag wires was very high.^18^ The qualitative inspection of the volume renderings shown in Figure 5, which highlight that the degradation of the Mg-4Ag wires proceeded inhomogeneously, supports this finding. A reason for the inhomogeneous degradation may be the nonuniform distribution of Ag in the wire. According to Liu et al.,^44^ the microstructure of Mg-*x*Ag alloys is strongly influencing the degradation behavior, with a larger number of precipitates leading to higher degradation rates.

The VL values of Mg-4Ag degraded at 37 °C are generally higher than those at RT and exhibit a steeper linear increase. This influence of the temperature is in accordance with previous studies on Mg-based alloys.^45^ In this case, DR of 9–12 mm a^–1^ are reached, which is higher than DR values of Mg-*x*Ag in the literature.^7,18,44,46−48^ This discrepancy in DR may be due to the different experimental conditions and their influences on the degradation behavior. Fluid flow is likely the most significant contributor to these differences,^20^ as well as the wire microstructure,^44^ temperature^45^ and pH buffering (see the Supporting Information).^49,50^ Overall, the presented experimental setup enables the observation of the degradation of Mg alloys under physiological conditions at early time points *in situ* (see Video S2).

### Microstructural Analysis Using Energy Dispersive X-ray Spectroscopy

To gain a better understanding of the materials microstructure and its chemical composition, the degradation layer and precipitates of the sample tested before with NFHT at 37 °C were analyzed thoroughly using scanning transmission electron microscopy (STEM) and EDX mapping.

**Figure 7:**
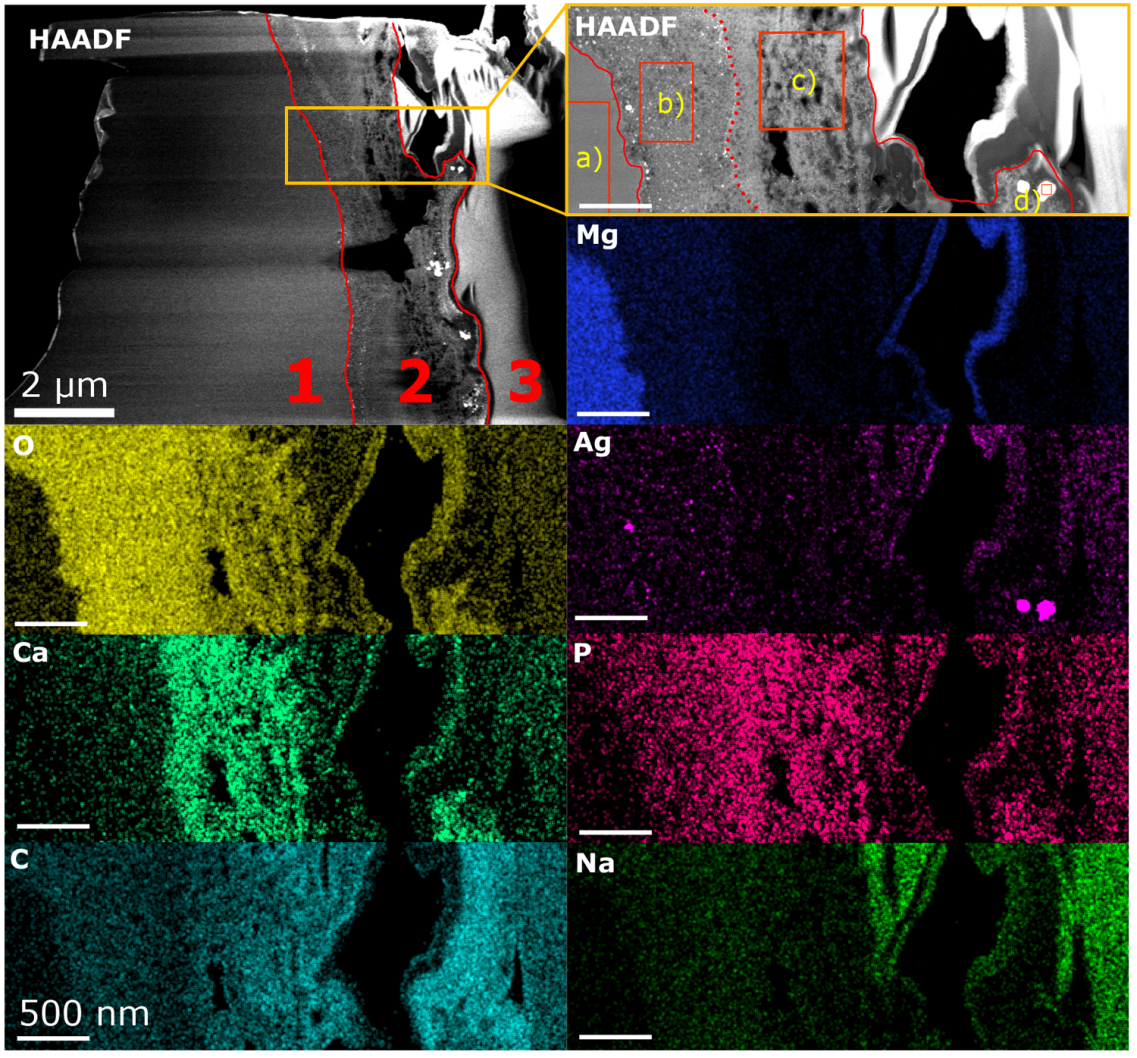
HAADF overview image of the thin Mg-4Ag foil originally degraded in SBF (37 °C, NFHT) for approximately 6 h. Red lines in the HAADF overview image separate bulk (1), degradation layer (2), and FIB protection layer (3). An orange rectangle indicates the sample area investigated with the aid of EDX with the magnified HAADF inset to the right. Chemical compositions (at.%) evaluated for the ROIs a–d, indicated in the HAADF inset, are summarized in Table 2. The dotted red line within the DL indicates the interface between two sub layers. The corresponding Mg, O, Ag, Ca, P, C, and Na distribution maps are shown below.

Figure 7 shows a high-angle annular dark field (HAADF) overview image of the structure observed in the TEM lamella prepared from this sample. The orange rectangle in the HAADF overview image indicates the area investigated with EDX, shown magnified in the right-hand side inset. Corresponding elemental distribution maps are presented below.

The average composition values of the individual regions of interest (ROIs), indicated as a–d in the HAADF inset in Figure 7, are listed in Table 2. ROI a stands for the non-degraded bulk material, containing mainly of Mg and Ag. Due to the presence of cracks and a high porosity of the degradation layer, the previously existing voids were enlarged during the thinning of the foil (e.g., black irregular hole in the middle of the DL) and the presence of a curtain effect within the bulk material (1) can be observed (horizontal lines with alternating brightness). In the bulk the Mg alloy is solid-solution annealed and no precipitates are visible in the HAADF images.

The observed degradation layer comprises two structurally distinct sub layers. The dotted red line in the HAADF inset marks the interface between the two sub layers. The inner sub layer, containing ROI b, is in direct contact with the bulk (non degraded sample material) and exhibits a rather compact but sponge-like structure with fine, homogeneously distributed pores. The outer sub layer is less concise, containing large, irregularly shaped, and nonhomogeneously distributed pores. Apart from changes in morphology, significant variations in Mg, O, and Ca concentrations are observed between the two sub layers.

From quantitative analysis of the EDX results in ROI b, a high content of Mg and O and low levels of Na, P, Cl, and Ca were revealed. The spongy sub layer structure and high O:Mg ratio, suggest that this inner sub layer consists predominantly of Mg(OH)_2_, which is consistent with the findings reported by Cihova et al.^51^

The chemistry of the outer layer, as exemplified by ROI c, reveals a decrease in Mg and O content with a simultaneous increase of the Ca and P amount. As the two latter elements originate from the immersive medium, this indicates that this part of the degradation layer was rather easily penetrated by the immersive medium, which was facilitated also by a higher layer porosity. Zeller-Plumhoff et al.^52^ present for pure Mg degraded in SBF the predicted predominant precipitation of hydroxyapatite Ca_5_(PO_4_)_3_OH. In addition, they show that small fractions of carbonates CaCO_3_ and MgCO_3_ can be expected at pH 7.4. Considering the presence of the elements in ROI c, it is thus assumed that a compound of hydroxapatite and Ca- and Mg-based carbonates formed during biodegradation.^53,54^

The degradation layer reveals the presence of unevenly distributed areas of bright appearance in the HAADF images, which correspond to the Ag-enriched areas on the corresponding EDX maps. The largest Ag-rich areas are mainly concentrated near the surface of the degradation layer, just beneath the carbon protection layer. One such area is marked in ROI (d) of the HAADF inset. Previous studies by Elen et al.^55^ and Liu et al.^44^ have reported the formation of precipitates in binary Mg-*x*Ag alloys, specifically identified as Mg_4_Ag and Mg_54_Ag_17_. Additionally, Tie et al.^56^ observed the presence of secondary phases, such as Mg_3_Ag and Mg_54_Ag_17_, during degradation processes. We observe the agglomeration of Ag, but the exact nature of its formation, whether as precipitates, secondary phases, or a combination of both, should be investigated in future experiments. Given the very high Ag:Mg ratio in ROI d, it is assumed that during the degradation process Ag agglomerates and diffuses through the degradation layer. Small Ag agglomerates can be observed in the inner sub layer, which may be trapped due to the porous structure. These agglomerates can diffuse through the outer sub layer and form larger agglomerates near the surface.

## Conclusion

A novel bioreactor-coupled flow-cell setup capable of biodegradation under physiological conditions while simultaneously allowing for *in situ* nanoimaging was developed. As a case study, the degradation of Mg-4Ag wires was investigated under the flow of SBF and autoregulation of physiological pH and temperature. We compared two full-field nanoimaging techniques, TXM and NFHT, and found that despite its longer postprocessing time, NFHT is better suited for the *in situ* imaging of biodegrading wires. The acquisition of VL and DR values by image segmentation of Mg-4Ag wires shows an increased degradation rate at higher temperatures and also the dynamic degradation behavior at very early time points, both according to the literature. The unique analytical data provided by *in situ* SRnanoCT revealed that the degradation of Mg-4Ag proceeds inhomogeneously. Since a resolution below 100 nm can be achieved, micro structural features like precipitates and pores may be observed. By utilizing STEM and EDX techniques, it is demonstrated that during degradation, two structurally distinct sub layers are formed. Moreover, it has been observed that the larger Ag agglomerates are predominantly located at the surface. To further our understanding of how precipitates affect degradation behavior, we are developing a correlative imaging approach that directly links nanoscopic CT data with HR(S)TEM and EDX data down to the atomic level. The unique data provided by correlative imaging can be used to aid the development of digital twins, which will be key in predicting and tailoring the biodegradation of Mg alloys in the future.

## Data Availability

The data sets generated and analyzed during the current study are available from the corresponding author on reasonable request.

## Supporting Information Available

Further details on the experimental setup, pH evolution of the bioreactor, and time lapse provided in the other Supporting Information files (PDF)
Video S1, flow-cell setup mounted on top of sample stage within the experimental hutch (MOV)
Graph of the pH evolution at the bioreactor (PDF)
Video S2, time lapse of a Mg-4Ag wire degrading in SBF (MP4)

## Supplementary Material

am3c04054_si_001.pdf

am3c04054_si_002.mov

## References

[ref1] Zeller-Plumhoff B.; Tolnai D.; Wolff M.; Greving I.; Hort N.; Willumeit-Römer R. Utilizing Synchrotron Radiation for the Characterization of Biodegradable Magnesium AlloysFrom Alloy Development to the Application as Implant Material. Adv. Eng. Mater. 2021, 23, 2100197 10.1002/adem.202100197.

[ref2] Su Z.; Nguyen T.-T.; Le Bourlot C.; Cadiou F.; Jamali A.; De Andrade V.; Franco A. A.; Demortière A. Towards a Local In situ X-ray Nano Computed Tomography under Realistic Cycling Conditions for Battery Research. Chem.: Methods 2022, 2, e202100051 10.1002/cmtd.202100051.

[ref3] Meyer Q.; Zeng Y.; Zhao C. In Situ and Operando Characterization of Proton Exchange Membrane Fuel Cells. Adv. Mater. 2019, 31, 1901900 10.1002/adma.201901900. 31373051

[ref4] Lu X.; Fernández M. P.; Bradley R. S.; Rawson S. D.; O’Brien M.; Hornberger B.; Leibowitz M.; Tozzi G.; Withers P. J. Anisotropic crack propagation and deformation in dentin observed by four-dimensional X-ray nano-computed tomography. Acta Biomater. 2019, 96, 400–411. 10.1016/j.actbio.2019.06.042. 31254684

[ref5] Li L.-B. Synchrotron Radiation Techniques: Watching Deformation-induced Structural Evolutions of Polymers. Chin. J. Polym. Sci. 2018, 36, 1093–1102. 10.1007/s10118-018-2169-9.

[ref6] Farhad F.; Smyth-Boyle D.; Zhang X.; Wallis I.; Panggabean D. Laboratory apparatus for in-situ corrosion fatigue testing and characterisation of fatigue cracks using X-ray micro-computed tomography. Fatigue Fract. Eng. Mater. Struct. 2018, 41, 2629–2637. 10.1111/ffe.12873.

[ref7] Zeller-Plumhoff B.; Helmholz H.; Feyerabend F.; Dose T.; Wilde F.; Hipp A.; Beckmann F.; Willumeit-Römer R.; Hammel J. U. Quantitative characterization of degradation processes in situ by means of a bioreactor coupled flow chamber under physiological conditions using time-lapse SRCT. Mater. Corros. 2018, 69, 298–306. 10.1002/maco.201709514.

[ref8] Witte F.; Eliezer A. In Degradation of Implant Materials; Eliaz N., Ed.; Springer: New York, 2012; pp 93–109.

[ref9] Zheng Y. F.; Gu X. N.; Witte F. Biodegradable metals. Mater. Sci. Eng., R 2014, 77, 1–34. 10.1016/j.mser.2014.01.001.

[ref10] Sekar P.; S N.; Desai V. Recent progress in in vivo studies and clinical applications of magnesium based biodegradable implants – A review. J. Magnesium Alloys 2021, 9, 1147–1163. 10.1016/j.jma.2020.11.001.

[ref11] Böstman O.; Pihlajamäki H. Routine implant removal after fracture surgery: A potentially reducible consumer of hospital resources in trauma units. J. Trauma 1996, 41, 846–849. 10.1097/00005373-199611000-00013. 8913214

[ref12] Wang J.-L.; Xu J.-K.; Hopkins C.; Chow D. H.-K.; Qin L. Biodegradable Magnesium-Based Implants in Orthopedics–A General Review and Perspectives. Adv. Sci. 2020, 7, 1902443 10.1002/advs.201902443. PMC7175270 32328412

[ref13] Zhang J.; Shang Z.; Jiang Y.; Zhang K.; Li X.; Ma M.; Li Y.; Ma B. Biodegradable metals for bone fracture repair in animal models: a systematic review. Regener. Biomater. 2021, 8, rbaa047 10.1093/rb/rbaa047. PMC7947587 33732493

[ref14] Chakraborty Banerjee P.; Al-Saadi S.; Choudhary L.; Harandi S. E.; Singh R. Magnesium Implants: Prospects and Challenges. Materials 2019, 12, 136 10.3390/ma12010136. 30609830 PMC6337251

[ref15] Withers P. J.; Bouman C.; Carmignato S.; Cnudde V.; Grimaldi D.; Hagen C. K.; Maire E.; Manley M.; Du Plessis A.; Stock S. R. X-ray computed tomography. Nat. Rev. Methods Primers 2021, 1, 18 10.1038/s43586-021-00015-4.

[ref16] Zeller-Plumhoff B.; Robisch A.-L.; Pelliccia D.; Longo E.; Slominska H.; Hermann A.; Krenkel M.; Storm M.; Estrin Y.; Willumeit-Römer R.; et al. Nanotomographic evaluation of precipitate structure evolution in a Mg-Zn-Zr alloy during plastic deformation. Sci. Rep. 2020, 10, 16101 10.1038/s41598-020-72964-x. 32999352 PMC7527343

[ref17] Zeller-Plumhoff B.; Laipple D.; Slominska H.; Iskhakova K.; Longo E.; Hermann A.; Flenner S.; Greving I.; Storm M.; Willumeit-Römer R. Evaluating the morphology of the degradation layer of pure magnesium via 3D imaging at resolutions below 40 nm. Bioact. Mater. 2021, 6, 4368–4376. 10.1016/j.bioactmat.2021.04.009. 33997513 PMC8111030

[ref18] Meyer S.; Wolf A.; Sanders D.; Iskhakova K.; Ćwieka H.; Bruns S.; Flenner S.; Greving I.; Hagemann J.; Willumeit-Römer R.; Wiese B.; Zeller-Plumhoff B. Degradation Analysis of Thin Mg-xAg Wires Using X-ray Near-Field Holotomography. Metals 2021, 11, 1422 10.3390/met11091422.

[ref19] Ogurreck M.; Wilde F.; Herzen J.; Beckmann F.; Nazmov V.; Mohr J.; Haibel A.; Müller M.; Schreyer A. The nanotomography endstation at the PETRA III Imaging Beamline. J. Phys.: Conf. Ser.J. Phys. 2013, 425, 182002 10.1088/1742-6596/425/18/182002.

[ref20] Gonzalez J.; Hou R. Q.; Nidadavolu E. P.; Willumeit-Römer R.; Feyerabend F. Magnesium degradation under physiological conditions – Best practice. Bioact. Mater. 2018, 3, 174–185. 10.1016/j.bioactmat.2018.01.003. 29744455 PMC5935771

[ref21] Silver S.; Phung L. T.; Silver G. Silver as biocides in burn and wound dressings and bacterial resistance to silver compounds. J. Ind. Microbiol. Biotechnol. 2006, 33, 627–634. 10.1007/s10295-006-0139-7. 16761169

[ref22] Tie D.; Feyerabend F.; Hort N.; Hoeche D.; Kainer K. U.; Willumeit R.; Mueller W. D. In vitro mechanical and corrosion properties of biodegradable Mg-Ag alloys. Mater. Corros. 2014, 65, 569–576. 10.1002/maco.201206903.

[ref23] Flenner S.; Kubec A.; David C.; Storm M.; Schaber C. F.; Vollrath F.; Müller M.; Greving I.; Hagemann J. Hard X-ray nano-holotomography with a Fresnel zone plate. Opt. Express 2020, 28, 37514 10.1364/OE.406074. 33379584

[ref24] Elsayed F. R.; Hort N.; Salgado Ordorica M. A.; Kainer K. U. Magnesium Permanent Mold Castings Optimization. Mater. Sci. Forum 2011, 690, 65–68. 10.4028/www.scientific.net/MSF.690.65.

[ref25] Nayeb-Hashemi A. A., Clark J. B., Eds. Phase Diagrams of Binary Magnesium Alloys; Monograph Series on Alloy Phase Diagrams; ASM International: Metals Park, OH, 1988; Vol. 4.

[ref26] Meyer S.; Wiese B.; Hort N.; Willumeit-Römer R. Characterization of the deformation state of magnesium by electrical resistance. Scr. Mater. 2022, 215, 114712 10.1016/j.scriptamat.2022.114712.

[ref27] Nienaber M.; Braatz M.; Ben Khalifa N.; Bohlen J. Property profile development during wire extrusion and wire drawing of magnesium alloys AZ31 and ZX10. Mater. Des. 2022, 224, 111355 10.1016/j.matdes.2022.111355.

[ref28] Haibel A.; Ogurreck M.; Beckmann F.; Dose T.; Wilde F.; Herzen J.; Müller M.; Schreyer A.; Nazmov V.; Simon M.; Last A.; Mohr J. Micro-and nano-tomography at the GKSS Imaging Beamline at PETRA III. Developments in X-Ray Tomography VII. 2010, 7804, 78040B 10.1117/12.860852.

[ref29] Flenner S.; Storm M.; Kubec A.; Longo E.; Döring F.; Pelt D. M.; David C.; Müller M.; Greving I. Pushing the temporal resolution in absorption and Zernike phase contrast nanotomography: enabling fast *in situ* experiments. J. Synchrotron Radiat. 2020, 27, 1339–1346. 10.1107/S1600577520007407. 32876609 PMC7467338

[ref30] Bohner M.; Lemaitre J. Can bioactivity be tested in vitro with SBF solution?. Biomaterials 2009, 30, 2175–2179. 10.1016/j.biomaterials.2009.01.008. 19176246

[ref31] Gürsoy D.; de Carlo F.; Xiao X.; Jacobsen C. TomoPy: A framework for the analysis of synchrotron tomographic data. J. Synchrotron Radiat. 2014, 21, 1188–1193. 10.1107/S1600577514013939. 25178011 PMC4181643

[ref32] Wittwer F.; Hagemann J.; Brückner D.; Flenner S.; Schroer C. G. Phase retrieval framework for direct reconstruction of the projected refractive index applied to ptychography and holography. Optica 2022, 9, 295–302. 10.1364/OPTICA.447021.

[ref33] Bruns S.; Stipp S.; Sørensen H. O. Looking for the Signal: A guide to iterative noise and artefact removal in X-ray tomographic reconstructions of porous geomaterials. Adv. Water Resour. 2017, 105, 96–107. 10.1016/j.advwatres.2017.04.020.

[ref34] Schindelin J.; et al. Fiji: An open-source platform for biological-image analysis. Nat. Methods 2012, 9, 676–682. 10.1038/nmeth.2019. 22743772 PMC3855844

[ref35] Moosmann J. moosmann/matlab (v1.0); 2021.

[ref36] Kovács A.; Schierholz R.; Tillmann K. FEI Titan G2 80–200 CREWLEY. JLSRF 2016, 2, A43 10.17815/jlsrf-2-68.

[ref37] Plis E. A.; Engelhart D. P.; Cooper R.; Johnston W. R.; Ferguson D.; Hoffmann R. Review of Radiation-Induced Effects in Polyimide. Appl. Sci. 2019, 9, 1999 10.3390/app9101999.

[ref38] Yan W.; Lian Y.-J.; Zhang Z.-Y.; Zeng M.-Q.; Zhang Z.-Q.; Yin Z.-Z.; Cui L.-Y.; Zeng R.-C. In vitro degradation of pure magnesium—the synergetic influences of glucose and albumin. Bioact. Mater. 2020, 5, 318–333. 10.1016/j.bioactmat.2020.02.015. 32181417 PMC7063336

[ref39] Saha P.; Roy M.; Datta M. K.; Lee B.; Kumta P. N. Effects of grain refinement on the biocorrosion and in vitro bioactivity of magnesium. Mater. Sci. Eng., C 2015, 57, 294–303. 10.1016/j.msec.2015.07.033. 26354267

[ref40] Walker J.; Shadanbaz S.; Kirkland N. T.; Stace E.; Woodfield T.; Staiger M. P.; Dias G. J. Magnesium alloys: Predicting in vivo corrosion with in vitro immersion testing. J. Biomed. Mater. Res., Part B 2012, 100B, 1134–1141. 10.1002/jbm.b.32680. 22331609

[ref41] Marco I; Myrissa A; Martinelli E; Feyerabend F; Willumeit-Romer R; Weinberg A.; Van der Biest O In vivo and in vitro degradation comparison of pure Mg, Mg-10Gd and Mg-2Ag: a short term study. Eur. Cells Mater. 2017, 33, 90–104. 10.22203/eCM.v033a07. 28197988

[ref42] Schön A.; Clarkson B. R.; Jaime M.; Freire E. Temperature stability of proteins: Analysis of irreversible denaturation using isothermal calorimetry. Proteins: Struct., Funct., Bioinf. 2017, 85, 2009–2016. 10.1002/prot.25354. PMC5706114 28722205

[ref43] Bechara B.; McMahan C. A.; Moore W. S.; Noujeim M.; Geha H.; Teixeira F. B. Contrast-to-noise ratio difference in small field of view cone beam computed tomography machines. J. Oral Sci. 2012, 54, 227–232. 10.2334/josnusd.54.227. 23047033

[ref44] Liu Z.; Schade R.; Luthringer B.; Hort N.; Rothe H.; Müller S.; Liefeith K.; Willumeit-Römer R.; Feyerabend F. Influence of the Microstructure and Silver Content on Degradation, Cytocompatibility, and Antibacterial Properties of Magnesium-Silver Alloys In Vitro. Oxid. Med. Cell. Longevity 2017, 2017, 1–14. 10.1155/2017/8091265. PMC5498933 28717409

[ref45] Esmaily M.; Shahabi-Navid M.; Svensson J.-E.; Halvarsson M.; Nyborg L.; Cao Y.; Johansson L.-G. Influence of temperature on the atmospheric corrosion of the Mg-Al alloy AM50. Corros. Sci. 2015, 90, 420–433. 10.1016/j.corsci.2014.10.040.

[ref46] Nidadavolu E. P. S.; Feyerabend F.; Ebel T.; Willumeit-Römer R.; Dahms M. On the Determination of Magnesium Degradation Rates under Physiological Conditions. Materials 2016, 9, 627 10.3390/ma9080627. 28773749 PMC5509045

[ref47] Marco I.; Feyerabend F.; Willumeit-Römer R.; Van der Biest O. Degradation testing of Mg alloys in Dulbecco’s modified eagle medium: Influence of medium sterilization. Mater. Sci. Eng., C 2016, 62, 68–78. 10.1016/j.msec.2016.01.039. 26952399

[ref48] Myrissa A.; Agha N. A.; Lu Y.; Martinelli E.; Eichler J.; Szakács G.; Kleinhans C.; Willumeit-Römer R.; Schäfer U.; Weinberg A.-M. In vitro and in vivo comparison of binary Mg alloys and pure Mg. Mater. Sci. Eng., C 2016, 61, 865–874. 10.1016/j.msec.2015.12.064. 26838918

[ref49] Xin Y.; Huo K.; Tao H.; Tang G.; Chu P. K. Influence of aggressive ions on the degradation behavior of biomedical magnesium alloy in physiological environment. Acta Biomater. 2008, 4, 2008–2015. 10.1016/j.actbio.2008.05.014. 18571486

[ref50] Zeng R.-C.; Hu Y.; Guan S.-K.; Cui H.-Z.; Han E.-H. Corrosion of magnesium alloy AZ31: The influence of bicarbonate, sulphate, hydrogen phosphate and dihydrogen phosphate ions in saline solution. Corros. Sci. 2014, 86, 171–182. 10.1016/j.corsci.2014.05.006.

[ref51] Cihova M.; Schmutz P.; Schäublin R.; Löffler J. F. Biocorrosion Zoomed In: Evidence for Dealloying of Nanometric Intermetallic Particles in Magnesium Alloys. Adv. Mater. 2019, 31, 1903080 10.1002/adma.201903080. 31486178

[ref52] Zeller-Plumhoff B.; Gile M.; Priebe M.; Slominska H.; Boll B.; Wiese B.; Würger T.; Willumeit-Römer R.; Meißner R. H. Exploring key ionic interactions for magnesium degradation in simulated body fluid – A data-driven approach. Corros. Sci. 2021, 182, 109272 10.1016/j.corsci.2021.109272.

[ref53] Brown P. W.; Constantz B. Hydroxyapatite and Related Material.; CRC Press: Boca Raton, FL, 1994; Vol. 368.

[ref54] Chen S.; Tu J.; Hu Q.; Xiong X.; Wu J.; Zou J.; Zeng X. Corrosion resistance and in vitro bioactivity of Si-containing coating prepared on a biodegradable Mg-Zn-Ca bulk metallic glass by micro-arc oxidation. J. Non-Cryst. Solids 2017, 456, 125–131. 10.1016/j.jnoncrysol.2016.11.011.

[ref55] Elen L.; Turen Y.; Ahlatci H.; Sun Y.; Unal M. Investigation of Microstructure, Mechanical and Corrosion Properties of Biodegradable Mg-Ag Alloys. Arch. Metall. Mater. 2022, 67, 889–900. 10.24425/amm.2022.139680.

[ref56] Tie D.; Feyerabend F.; Hort N.; Hoeche D.; Kainer K. U.; Willumeit R.; Mueller W. D. In vitro mechanical and corrosion properties of biodegradable Mg-Ag alloys: In vitro mechanical and corrosion properties of Mg-Ag alloys. Mater. Corros. 2014, 65, 569–576. 10.1002/maco.201206903.

