# Supplementary material for: Development of a Bioreactor-Coupled Flow-Cell Setup for 3D In Situ Nanotomography of Mg Alloy Biodegradation
Source: ACS Appl Mater Interfaces. 2023 Jul 17;15(29):35600–10. doi: 10.1021/acsami.3c04054 (PMC10375473; doi:10.1021/acsami.3c04054)
Supplement: Supplementary file 1 — am3c04054_si_001.pdf [file am3c04054_si_001.pdf]

Development of a bioreactor-coupled flow-cell setup for 3D in situ nanotomography of Mg alloy biodegradation

Jan Reimers,[*,†,‡,⊥] Huu Chánh Trinh,[†,⊥] Björn Wiese,[†] Sebastian Meyer,[†] Jens Brehling,[¶] Silja Flenner,[¶] Johannes Hagemann,[§] Maximilian Kruth,[‡] Lidia Kibkalo,[‡] Hanna Ćwieka,[†] Birte Hindenlang,[†] Marta Lipinska-Chwalek,[‡] Joachim Mayer,[‡,‖] Regine Willumeit-Römer,[†] Imke Greving,[¶] and Berit Zeller-Plumhoff[*,†]

†*Institute of Metallic Biomaterials, Helmholtz-Zentrum Hereon, Max–Planck–Str. 1, 21502 Geesthacht, Germany*

‡*Ernst Ruska-Centre for Microscopy and Spectroscopy with Electrons, Forschungszentrum Jülich GmbH, 52425 Jülich, Germany*

¶*Institute of Materials Physics, Helmholtz-Zentrum Hereon, Max–Planck–Str. 1, 21502, Germany*

§*CXNS—Center for X-ray and Nano Science, Deutsches Elektronen-Synchrotron DESY, Notkestraße 85, 22607 Hamburg, Germany*

‖*Central Facility for Electron Microscopy, RWTH Aachen University, Ahornstraße 55, 52074 Aachen, Germany*

⊥*These authors contributed equally as main authors to this work*

E-mail: jan.reimers@hereon.de; berit.zeller-plumhoff@hereon.de

Supporting Information

SI-1: Experimental setup

The video SI-1 shows the second generation flow-cell setup mounted on top of the sample stage within the experimental hutch. On the right side the optics for NFHT imaging are installed. The direction of the beam goes from right to left side. The wires and tubes connecting the flow-cell and the bioreactor are inside the black braided sleeve. The braided sleeve is positioned between two guiding plates which ensure that the optics cannot be touched when the flow cell is rotated.

SI-2: pH evolution

During experiments with the first generation flow-cell setup, the pH was adjusted manually while an automatic system was used during the second generation. During the beamtime, it was not possible to automatically control the pH within the bioreactor using CO_2. Hence, the pH had to be adjusted manually by interrupting the imaging process periodically. The figure SI-2 shows the medium's pH value in the bioreactor over time during the *in situ* SRnanoCT of the Mg-4Ag wires. It can be seen that the pH had to be reduced back down to about 7.4 several times before the pH reaches levels above 7.6. The CO_2 valve was left open which introduced a constant amount of CO_2 into the medium. Only sometimes the pH rising effect of the degradation was successfully counteracted.

After the beamtime, it was revealed that the control unit was defective and repairs by the manufacturer MDX Biotechnik International GmbH were necessary. The switching output for the CO_2 valve was broken and after the repairs the switching output for the acid pump is now used to control the CO_2 valve. After the repairs, a degradation test using a Mg-4Ag wire with SBF as flow medium was conducted without imaging. In the figure SI-2, the pH value is shown during the 10-hour-long degradation experiment. The pH value zigzags around the target pH of 7.4 while never exceeding a value of 7.45. The average pH value is 7.40 with

the highest value being 7.45 and lowest 7.32. The increase in pH is probably caused by the outgassing of CO_2 and alkalic degradation products. The upper pH threshold is determined by the so-called "Dead band" value which determines when new CO_2 is introduced into the medium containing bioreactor. In this case, the Dead band value was set to 0.05 meaning that at 7.45 CO_2 is injected into the bioreactor. The control unit closes the CO_2 valve when the pH decreases to 7.425. However, there is a delay between the shut off and the actual stop of CO_2 gas flow into the bioreactor. Hence, the pH is lowered beyond 7.4. All in all, the automatic pH adjustment functions reliably and can ensure a physiological pH within the bioreactor. However, as the pH adjusted medium flows to the flow-cell, its pH might change due to outgassing of the CO_2. The silicone rubber tubes in which the medium flows are known to have a relatively high gas permeability so it is likely that the actual pH at the flow-cell is higher than 7.4.

SI-3: Degradation time lapse

In SI-3 tomographic slices of sample three (37 °C, NFHT) were taken at each time point (according to Figure 6) at approximately the same sample position.
