# Supplementary figures and images for: Development of a Bioreactor-Coupled Flow-Cell Setup for 3D In Situ Nanotomography of Mg Alloy Biodegradation
Source: ACS Appl Mater Interfaces. 2023 Jul 17;15(29):35600–10. doi: 10.1021/acsami.3c04054 (PMC10375473; doi:10.1021/acsami.3c04054)

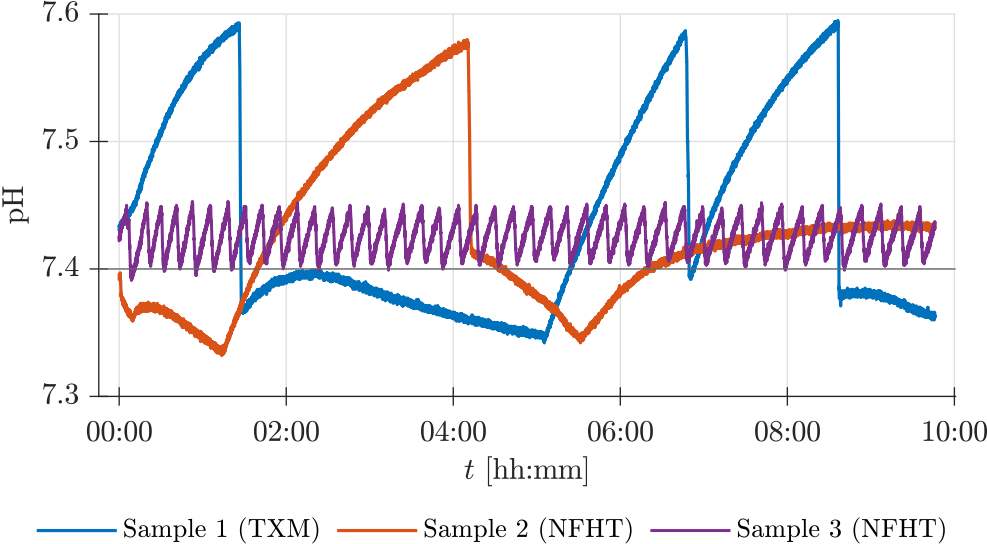

Supplement: Supplementary file 3 — am3c04054_si_003.pdf [file am3c04054_si_003.pdf]
